# Pediatric AKI in the real world: changing outcomes through education and advocacy—a report from the 26th Acute Disease Quality Initiative (ADQI) consensus conference

**DOI:** 10.1007/s00467-023-06180-w

**Published:** 2023-11-07

**Authors:** Theresa Mottes, Shina Menon, Andrea Conroy, Jennifer Jetton, Kristin Dolan, Ayse Akcan Arikan, Rajit K. Basu, Stuart L. Goldstein, Jordan M. Symons, Rashid Alobaidi, David J. Askenazi, Sean M. Bagshaw, Matthew Barhight, Erin Barreto, Benan Bayrakci, O. N. Bignall Ray, Erica Bjornstad, Patrick Brophy, Jennifer Charlton, Rahul Chanchlani, Andrea L. Conroy, Akash Deep, Prasad Devarajan, Dana Fuhrman, Katja M. Gist, Stephen M. Gorga, Jason H. Greenberg, Denise Hasson, Emma Heydari, Arpana Iyengar, Catherine Krawczeski, Leslie Meigs, Catherine Morgan, Jolyn Morgan, Tara Neumayr, Zaccaria Ricci, David T. Selewski, Danielle Soranno, Natalja Stanski, Michelle Starr, Scott M. Sutherland, Jordan Symons, Marcelo Tavares, Molly Vega, Michael Zappitelli, Claudio Ronco, Ravindra L. Mehta, John Kellum, Marlies Ostermann, the ADQI 26 workgroup

**Affiliations:** 1 Division of Nephrology, Robert Lurie Children’s Hospital of Chicago, Ann &, Chicago, IL USA; 2 Department of Pediatrics, Seattle Children’s Hospital, University of Washington School of Medicine, Seattle, WA USA; 3 Department of Pediatrics, Pediatric Infectious Disease and Global Health, Indiana University School of Medicine, Indianapolis, IN USA; 4 Section of Pediatric Nephrology, Department of Pediatrics, Medical College of Wisconsin, Milwaukee, WI USA; 5 Section of Critical Care Medicine, Department of Pediatrics, Baylor College of Medicine, Houston, TX USA; 6 Section of Critical Care Medicine and Section of Nephrology, Department of Pediatrics, Baylor College of Medicine, Houston, TX USA; 7 Division of Critical Care Medicine, Department of Pediatrics, Robert Lurie Children’s Hospital of Chicago, Northwestern University Feinberg School of Medicine, Ann &, Chicago, IL USA; 8 Division of Pediatric Nephrology, Department of Pediatrics, Cincinnati Children’s Hospital Medical Center, Cincinnati, OH USA

**Keywords:** Acute kidney injury, Advocacy, Education, Education Design Research (EDR)

## Abstract

**Background:**

Acute kidney injury (AKI) is independently associated with increased morbidity and mortality across the life course, yet care for AKI remains mostly supportive. Raising awareness of this life-threatening clinical syndrome through education and advocacy efforts is the key to improving patient outcomes. Here, we describe the unique roles education and advocacy play in the care of children with AKI, discuss the importance of customizing educational outreach efforts to individual groups and contexts, and highlight the opportunities created through innovations and partnerships to optimize lifelong health outcomes.

**Methods:**

During the 26th Acute Disease Quality Initiative (ADQI) consensus conference, a multidisciplinary group of experts discussed the evidence and used a modified Delphi process to achieve consensus on recommendations on AKI research, education, practice, and advocacy in children.

**Results:**

The consensus statements developed in response to three critical questions about the role of education and advocacy in pediatric AKI care are presented here along with a summary of available evidence and recommendations for both clinical care and research.

**Conclusions:**

These consensus statements emphasize that high-quality care for patients with AKI begins in the community with education and awareness campaigns to identify those at risk for AKI. Education is the key across all healthcare and non-healthcare settings to enhance early diagnosis and develop mitigation strategies, thereby improving outcomes for children with AKI. Strong advocacy efforts are essential for implementing these programs and building critical collaborations across all stakeholders and settings.

## Introduction

Understanding of pediatric acute kidney injury (AKI) continues to advance, with increasing recognition of different phenotypes and underlying pathophysiology. Management of AKI remains mostly supportive, aimed at early identification and interventions to mitigate progression. Lack of AKI awareness among healthcare workers and insufficient knowledge in the public of risk factors, signs, symptoms, and complications of AKI impede early identification. Inappropriate resources in different clinical settings and limited governmental/institutional support challenge the ability to improve AKI care through education and/or quality improvement. The 26th Acute Disease Quality Initiative (ADQI) consensus conference was convened to improve clinical care and identify the research agenda in pediatric AKI. This article addresses the roles education and advocacy play in improving care and outcomes related to AKI in children. We ask and answer three key questions:

- Question 1: What unique roles do education and advocacy play in advancing AKI care for children?
- Question 2: How do we customize AKI education and advocacy efforts for diverse groups and settings?
- Question 3: What innovations and partnerships will raise awareness, overcome barriers, and advocate for optimal lifelong outcomes in AKI?

## Methods

The 26th ADQI consensus conference was held over 3 days in Napa, USA, in November 2021, and included an interdisciplinary group of clinicians and researchers from North and South America, Africa, Asia, and Europe. The first ADQI conference with a completely pediatric focus, this group included 47 multidisciplinary experts from pediatric nephrology, pediatric and adult critical care, pharmacy, epidemiology, health services research, pediatric nephrology nursing, nutrition, and patients, as previously described [1]. This meeting followed the established ADQI process [2]. A detailed description of the ADQI methodology is available at www.adqi.net. The broad objective of ADQI is to provide expert-based statements and a summary of current knowledge for use by clinicians and researchers according to ADQI’s professional judgment and to identify evidence gaps to establish research priorities.

The Education and Advocacy workgroup sought to develop consensus statements to enhance education efforts and to provide a framework for advocacy to improve outcomes of children with and at risk for AKI. The workgroup included clinicians and researchers, as well as an AKI survivor, and an expert in advocacy and social determinants of health. The consensus-building process employed an objective review of articles identified through a PubMed search. The results of this consensus conference are not based on a formal systematic review process; rather, a modified Delphi method was used to reach consensus, using evidence-based literature when possible, with the goal of addressing the key questions and articulating a research agenda to address knowledge gaps. A summary of the consensus conference has been previously published [1]. We describe our workgroup’s consensus findings, as well as detailed recommendations to be used as a framework for the advancement of pediatric AKI care through educational and advocacy approaches.

## Results

A critical recommendation of the consensus panel was a need to improve pediatric AKI education and advocacy efforts:

Given the adverse immediate and lifelong outcomes for children with AKI, education and advocacy are essential, starting with the patient and family and expanding across health care teams, systems, and communities.

We thus sought to answer three questions developed during the ADQI consensus conference.

### Question 1: What unique roles do education and advocacy play in advancing AKI care for children?

#### Consensus statements

1a. Given the significant short- and long-term risks to patients, and the high burden of AKI on healthcare systems, greater education about AKI is necessary for all members of the healthcare system.

1b. In the absence of easily recognizable signs and symptoms and the lack of specific treatment for AKI, education and advocacy are essential to improve understanding (awareness) and recognition of AKI*.*

Despite three decades of intensified research, AKI-related morbidity and mortality rates remain unacceptably high. AKI may lack clinical signs or symptoms and can occur in any patient. Therefore, a critical step in addressing the AKI epidemic is robust education aimed at improving awareness of the impact of AKI and recognition of risk factors. The first contact with the healthcare system for a patient with AKI is generally in the primary care setting, emergency room, or inpatient wards by non-nephrologists. The healthcare professional may be a medical student, resident, attending physician, advanced practice provider, nurse, community health care worker, or pharmacist. Each of these levels of direct patient care offers opportunities to improve AKI recognition and mitigate AKI risks [3].

AKI awareness remains variable across different healthcare settings, contributing to disparities in outcomes [4]. AKI often goes unrecognized in the community and outside the intensive care setting in the hospital [5]. Community-acquired AKI occurred in 1.5% of emergency room encounters and 37% of AKI was missed by the emergency or inpatient clinician in those admitted [6]. In a survey evaluating AKI education and knowledge among nursing staff in rural South Africa, only 37.8% reported confidence in managing a patient with AKI; 97% reported wanting additional training. Similarly, 61% of nurses across 11 districts reported never receiving training on AKI, and one-third of health care workers were unaware of dialysis availability at the National Referral Hospital in Malawi [7]. Given how frequently AKI goes unrecognized, developing AKI education specific to non-nephrologists is a critical step for improving AKI care [8].

Improving awareness and early identification of AKI must focus on improving clinical care. In Malawi, following a nurse-led educational intervention that included a comprehensive classroom and ward-based teaching on AKI, there was a significant improvement in the healthcare workers’ attitudes towards detecting or managing patients with suspected AKI with concomitant improvements in care processes (e.g., completion of fluid charts and recording of urine output) [9]. The use of checklists, standardized screening, and care protocols (“care bundles”) in both inpatient and outpatient settings enhance detection and identification of patients with or at risk for AKI [10]. Clinical assessment tools deployed in three low-resource countries predict KDIGO stage 3 AKI [11]. Educational interventions at teaching hospitals in the UK, designed to increase understanding of AKI, demonstrated increased physician awareness based on self-reported management of patients and comparison of AKI knowledge assessed on pre- and post-education questionnaires [12]. In another UK study, electronic alerts deployed at a teaching hospital not only raised awareness of AKI but improved outcomes, leading to less progression to higher stages of AKI, fewer emergency re-admissions, and a reduction in mortality [13]. A pediatric multi-center intervention to raise awareness of the impact of nephrotoxic medications on the development of AKI led to decreased AKI rates [14]. More studies are needed, particularly in the pediatric age group.

**Fig. 1:**
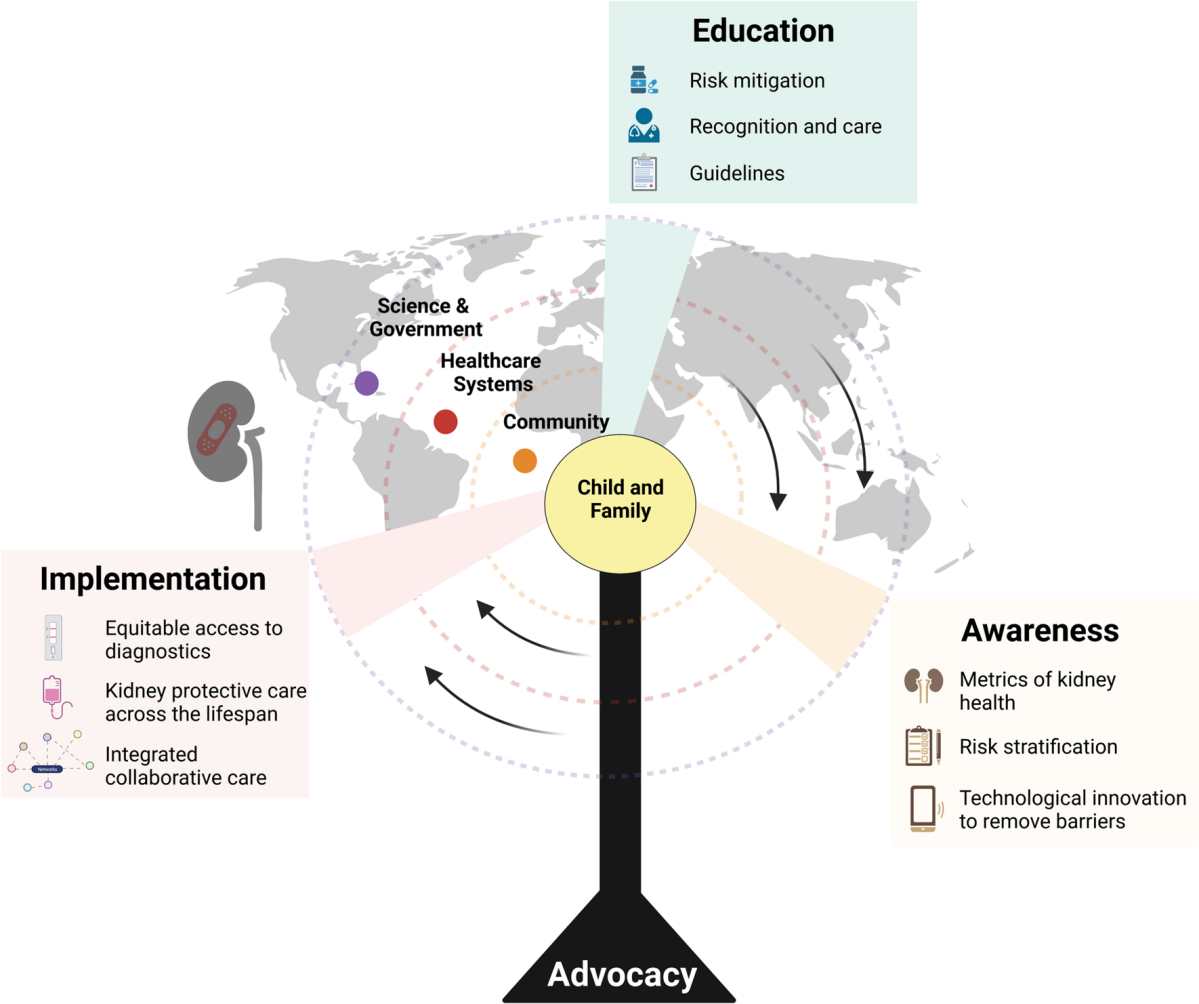
Effective acute kidney injury (AKI) education programs center the child and family (yellow circle at center) and expand across health care teams, systems, and communities with champions embedded at every level (community (orange), healthcare systems (red), science and government (purple)), all supported by the pillar of advocacy

Improving education and awareness about kidney disease requires strong advocacy efforts to unify the different constituents who participate in AKI prevention, early recognition, and treatment (Fig. 1). These constituents include patients and family members, community members, physicians within and outside of nephrology, allied healthcare professionals, industry affiliates, government, and non-governmental organizations. Each has unique skills, vantage points, and priorities within the healthcare team that can be harnessed in mitigating pediatric AKI. Champions within each of these groups can highlight the relevance of AKI to their own constituents to advocate for and obtain the human and financial resources needed to grow and sustain these efforts.

#### Research recommendations

1. Future work should emphasize strong AKI-related advocacy efforts among all constituents.
2. Future work should focus on the recognition and improvement of clinical care at the bedside through more accurate detection and identification of patients with or at risk for AKI.
3. Future work should seek to bring knowledge of AKI and its risks to the general population.

### Question 2: How do we customize AKI education and advocacy efforts for diverse groups and settings?

#### Consensus statement

2. Given the complexity of the healthcare system and multiple stakeholders involved, AKI education requires a comprehensive, multi-disciplinary approach with AKI champions embedded at every level, with appropriate consideration of local context.

AKI education may be accomplished through numerous methods, tailored for a variety of learners, at the micro (e.g., teaching individual learners, training program curriculum, teaching modules, alert systems for clinicians) and macro (e.g., healthcare system, community-wide educational programs, governmental policy) level. Recognizing the integral role that families provide in child health and development, and that most AKI develops in the community, AKI education programs should center on children and their families. Educating and empowering patients and families allow them to participate in AKI prevention at home, reducing the burden of undetected AKI in children. During illness, patients and families may not be aware of an AKI event or the associated long-term risks [15, 16]; patient and family education can yield healthy lifestyle modification and may improve follow-up, limiting repeat AKI events. Patients and families require diverse, adaptable, and multifaceted educational tools in a variety of primary languages, levels of health literacy, and cultural contexts. With increasing recognition of diminished health-related quality of life in AKI survivors [17, 18] and worse neurodevelopmental outcomes among pediatric AKI survivors [19–22], there is a need for evidence on how to provide appropriate psychosocial support to AKI survivors, tailored to the unique developmental needs of the child.

AKI affects the outcomes of many conditions and illnesses managed primarily by non-nephrologists (e.g., congenital heart disease undergoing surgical care, oncologic disease, and critical illness in neonates). Complex healthcare systems with highly specialized care teams can lead to overly routinized thinking that may limit recognition of off-target clinical concerns like AKI. Such siloed approaches could be overcome with focused education to enhance collaboration. Educational and advocacy efforts can be tailored to each discipline and for clinicians, bedside nurses, and other interdisciplinary team members (e.g., ICU dietitians and pharmacists) to optimize relevance for each. In lower income settings and rural areas, community health extension workers may be the first point of contact for patients, and much of the initial recognition and management of AKI is undertaken by them.

We recommend developing clear and consistent objectives for AKI education along with methods to evaluate their efficacy. These objectives should be developed with community engagement incorporating suggestions from the public, patients and caregivers, and community healthcare workers. For practitioners, AKI education must be a core competency [8, 23]. In the UK, the Academy of Royal Medical Colleges has codified the knowledge, skills, and behaviors required for safe and effective care of a patient with AKI [24]. This approach can be adapted for education of all healthcare professionals at all levels and should be integrated as part of continuing medical education. The Latin American Society of Nephrology and Hypertension created two online courses, one for nephrologists and one for primary care physicians, to improve AKI awareness [25]. Learning objectives for the two courses were specific to the needs of each target audience. There was a mean knowledge gain of 36% seen with pre- and post-course tests. Both groups expressed high rates of satisfaction with the activity and commitment to improve their clinical practice. With the implementation of educational programs will come the need to monitor knowledge gain, improvement in quality of care, and differences in patient outcomes.

While we recognize the importance of AKI education, there are limited data on how most effectively to structure such programs. Developing meaningful educational programs requires thoughtful preparation, innovative design, and a robust iterative review process. These are key principles in Education Design Research (EDR), a pragmatic and participatory approach for solving educational problems within the authentic, complex educational setting [26, 27]. For AKI education, given its broad and diverse spectrum of participants and stakeholders, local problem analysis is imperative. Identifying participants and examining barriers (e.g., time and competing priorities) to enhance awareness and knowledge is a necessary first step. This can be done with qualitative surveys, needs assessments, literature reviews, reviews of societal guidelines, and assessment of existing educational programs. Once this process is complete, design and implementation of educational systems can occur alongside continuous evaluation and iteration of each program. Online simulation modules for nursing students to recognize patient deterioration [28] and using virtual patients and concept mapping software to teach clinical reasoning skills to medical students are examples of EDR implementation [29]. EDR is also utilized outside of medical education programs highlighting the ability to apply these principles in diverse settings.

The nephrology community can learn from successful initiatives for other conditions (e.g., diabetes, HIV) where education has been shown to improve outcomes including clinical, financial, and patient quality of life. A multisector model with public/private partnership, community support, and use of local resources has shown success in achieving various Millennium Development Goals (MDGs) by increasing awareness and intervention [30]. Following the MDGs, the Sustainable Development Goals (SDGs) were established to provide a more holistic set of goals that promote healthy and sustainable societies; these have been applied to kidney health [31]. Multisector models with public/private partnerships that engage United Nations agencies, governments, businesses, and local community partners have been successful in achieving various MDGs by increasing awareness and intervention. The nephrology community must engage international partners working on global health targets to integrate AKI prevention in the programs. These efforts should be driven by the nephrology community through organizations like the International Pediatric Nephrology Association and the International Society of Nephrology through programs such as the AKI 0 by 25 program and engaging stakeholders at the policy level, including the World Health Organization.

Lessons from other health programs can inform the design of an AKI-focused curriculum [32]. Traditionally such programs were designed and taught by those from high-resource countries, with limited input from and consideration of diverse global perspectives and practices. Recently, the Synthesis and Translation of Research and Innovations from Polio Eradication (STRIPE) consortium described its collaborative process of developing content and curriculum for an international course, along with recommendations for future similar efforts [33]. The course designed by the STRIPE consortium has an online component (massive open online course (MOOC)) along with in-person courses taught by local experts that could reach students in a variety of public health contexts across the world.

While MOOCs are effective in reaching a global audience, their production can be resource intensive. With trainees increasingly using social media platforms for free open-access medical education [34], efforts are needed to develop online AKI educational resources that increase access to evidence-based information appropriate for pediatric populations. Nephrology-specific educational resources in the form of games, online journal clubs, and interactive learning are making education available globally 24 h a day [35, 36]. Such platforms can be harnessed to increase access to education outside of traditional classrooms, conferences, and local institutions.

#### Research recommendations

1. Future work should begin with a thorough, in-depth problem analysis and evaluation of the educational problem which is key to successful implementation and future systematic change of education programs.
2. Future work should include creating AKI education programs that are applicable to various settings and resources and adaptable to local needs and knowledge gaps.

### Question 3: What innovations and partnerships will raise awareness, overcome barriers, and advocate for optimal lifelong outcomes in AKI?

#### Consensus statement

3. AKI awareness, prevention, and mitigation requires coordinated efforts to develop effective tools that will preempt and screen for AKI and provide guidance for care acutely and long-term. In partnership with industry, government, non-governmental organizations, and professional societies, these tools must leverage current and advancing technology to yield efficient and equitable care regardless of local resources*.*

While AKI-related education may increase awareness, timely recognition of AKI is dependent on the availability of diagnostic tools for AKI risk and recovery. Effectiveness of tools may depend on clinical context and available resources; increasing partnerships between industry, governmental, and non-governmental stakeholders will focus attention and resources to improve AKI diagnosis. A survey of global health information registries in 154 countries showed that while 57% of countries had transplant registries (range 15–100% by ISN region), 8% of countries had AKI registries and 4% had programs in place to detect AKI [37]. Partnerships to establish AKI registries with support from governmental and non-governmental stakeholders will facilitate data collection and inform local needs in addressing priority AKI populations. AKI advocates are needed to reach out to these entities through effective campaigns and persuade them to allocate resources to these efforts. Toolkits created and shared by content experts would support these local efforts [8].

In low-resource settings, inadequate training, shortage of healthcare workers, and lack of awareness by health care teams, governments, and the public are major barriers to recognizing AKI and understanding its significance as a health problem [38]. Government buy-in is crucial to ensure adequate resources to support the education of healthcare workers and the provision of diagnostic aids and basic therapies. Programs in low and lower middle-income countries can lose trained staff who leave to work in higher-resourced areas [39]. Appropriate support from the local government may encourage trained staff to remain in their own countries with the confidence that they may be able to develop successful programs.

Partnership with industry can be leveraged across multiple domains, whether it is the development and distribution of newer diagnostic tools, or harnessing information technology to educate, monitor or follow up patients with AKI. Limited diagnostic and treatment facilities in health centers are reported as a significant barrier in global AKI care [38]. Lack of laboratory infrastructure leads to substantial inequities in access to AKI diagnostics and further accentuates adverse healthcare outcomes from delayed diagnosis of AKI. Access to creatinine testing may be limited or unavailable in outpatient, rural, or low-income settings. Point of care creatinine can serve where central laboratory testing facilities may not be available [11]. Biomarkers of structural kidney injury could help identify children in need of additional monitoring in-hospital and also risk-stratify children who may benefit from additional clinical monitoring to assess kidney recovery [40]. Urine neutrophil gelatinase-associated lipocalin (NGAL) has been adapted to a urine dipstick test which correlates well with reference assays and has been used for diagnosis of pediatric AKI in Africa [41, 42]. Point-of-care tests that can be used in community settings to identify children based on creatinine or alternative indicators of AKI (e.g., saliva urea nitrogen, urine NGAL dipsticks) have potential to overcome barriers related to accessible, affordable, and equitable AKI diagnostics.

Increasing global interconnectedness raises opportunities to leverage technology to support equitable diagnosis of AKI. Technological approaches to facilitate AKI diagnosis and follow-up must be adapted based on the availability and integration of health information technology within health systems. Telemedicine can establish linkages across settings, both within and between countries, to ensure that patients in remote, rural, or low-income settings have access to specialized care and appropriate follow-up without necessarily requiring in-person referral [11]. This could improve clinical care, optimize follow-up, and expand education to health care workers in more remote settings.

In settings where advanced health information technology is limited, there is often relatively easier access to mobile health technology where apps could be used to integrate AKI risk assessment as part of the triage process for the sick child. A precision public health approach has been used to improve post-discharge outcomes in children following sepsis events in Uganda [43]. The program, called Smart discharges, uses risk prediction to identify at-risk children, counsel parents at discharge about potential complications and when to seek care, and connect high-risk children to community health centers and healthcare workers for follow-up. It can be used as a stand-alone mobile application or be integrated into existing electronic health platforms. Such a program can be adapted to include AKI risk factors and danger signs and represents an opportunity to leverage partnerships to improve AKI risk prediction, identification, and follow-up care in settings where formal systems are lacking.

In resource-sufficient settings that utilize electronic health records, clinical decision support systems could be adapted to prompt AKI assessment based on presenting signs and automated to detect AKI events and recommend additional monitoring and follow-up.

#### Research recommendations

1. Future work should be focused on using iterative testing to refine diagnostic and therapeutic innovations.
2. Future work should expand connections between healthcare, industry, government and other groups to bring AKI prevention, diagnosis, and care to all communities.

## Conclusions

**Fig. 2:**
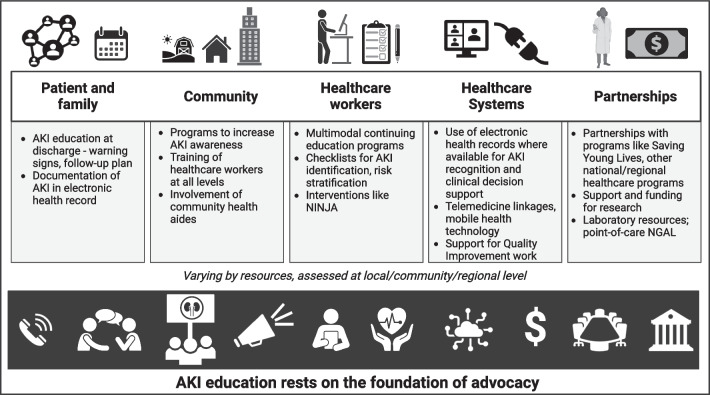
Education to improve acute kidney injury (AKI) awareness and recognition in children rests on the foundation of advocacy and must engage key stakeholders in developing multidisciplinary, context-appropriate, and customized approaches to improve AKI care globally and across all resource settings. NINJA, Nephrotoxic injury negated by just-in-time action program; NGAL, neutrophil gelatinase-associated lipocalin. Permission to use content, including icons, templates, and other original artwork, granted by BioRender

AKI is an increasingly important public health concern. These consensus statements emphasize that high-quality care for patients with AKI begins in the community with education and awareness campaigns to identify patients at risk for AKI (Fig. 2). Education is the key across all healthcare and non-healthcare settings to improve early diagnosis and implement mitigation strategies, thereby improving outcomes for children with AKI. More research is needed to design educational programs with clear and consistent objectives for AKI learning along with methods to address and evaluate the efficacy of implementation of these objectives. Education is essential but not sufficient; collaboration between health care teams, medical systems, and institutional organizations is necessary to develop AKI identification tools which leverage modern technology and are adaptable to any clinical venue, community, or resource environment. To realize these programs, strong advocacy efforts with clear messaging relevant to various stakeholders are needed to overcome financial, time, infrastructure, and human resource limitations. Developing strong communities of AKI champions may be the key to overcoming short- and long-term effects of this potentially devastating clinical syndrome and improving outcomes for children around the world.
